# A microfabricated multi-compartment device for neuron and Schwann cell differentiation

**DOI:** 10.1038/s41598-021-86300-4

**Published:** 2021-03-29

**Authors:** Eleonora De Vitis, Velia La Pesa, Francesca Gervaso, Alessandro Romano, Angelo Quattrini, Giuseppe Gigli, Lorenzo Moroni, Alessandro Polini

**Affiliations:** 1 CNR NANOTEC – Institute of Nanotechnology, Campus Ecotekne, via Monteroni, 73100 Lecce, Italy; 2 Dipartimento di Matematica e Fisica E. de Giorgi, Università Del Salento, Campus Ecotekne, via Monteroni, 73100 Lecce, Italy; 3 Division of Neuroscience, Institute of Experimental Neurology, IRCCS San Raffaele Scientific Institute, 20132 Milan, Italy; 4 Complex Tissue Regeneration, Maastricht University, Universiteitssingel 40, Maastricht, 6229 ER The Netherlands

**Keywords:** Biomedical engineering, Biotechnology

## Abstract

Understanding the complex communication between different cell populations and their interaction with the microenvironment in the central and peripheral nervous systems is fundamental in neuroscience research. The development of appropriate in vitro approaches and tools, able to selectively analyze and/or probe specific cells and cell portions (e.g., axons and cell bodies in neurons), driving their differentiation into specific cell phenotypes, has become therefore crucial in this direction. Here we report a multi-compartment microfluidic device where up to three different cell populations can be cultured in a fluidically independent circuit. The device allows cell migration across the compartments and their differentiation. We showed that an accurate choice of the device geometrical features and cell culture parameters allows to (1) maximize cell adhesion and proliferation of neuron-like human cells (SH-SY5Y cells), (2) control the inter-compartment cell migration of neuron and Schwann cells, (3) perform long-term cell culture studies in which both SH-SY5Y cells and primary rat Schwann cells can be differentiated towards specific phenotypes. These results can lead to a plethora of in vitro co-culture studies in the neuroscience research field, where tuning and investigating cell–cell and cell–microenvironment interactions are essential.

## Introduction

The nervous system is, probably, the most complex part of an animal organism, responsible of actions and sensory information by transmitting signals to and from different parts of the body. The two broad classes of cells in the nervous system are neurons and glial cells, present in approximately equal number and interconnected via complex circuitries. Glial cells, also known as the “sleeping giant” of neuroscience, are non-neuronal cells involved in different neuronal activities (i.e. maintenance of homeostasis, myelin formation, supplying nutrients and oxygen and defense from pathogens)^1^. Neurons are electrically excitable cells that can communicate with other cells via specialized connections called synapses. A typical neuron consists of a cell body called soma, dendrites that typically branch profusely and a single axon that reaches different tissues and targets with specific chemical and physical features.

In order to investigate the basic functions of the nervous system and the pathogenic mechanisms of neurological disorders, it is of paramount importance to understand how molecular, physical and biochemical cues modulate the neuronal and non-neuronal cell dynamics^2^. In vivo, all these signals vary spatially and temporally. Conventional neuron cultures (i.e. petri dish) do not allow to reproduce in vitro the extracellular environment specific of the in vivo systems. Moreover, in these systems neurons are cultured in homogeneous media, randomly seeded, and their interactions depend on distribution and proximity, making the signals difficult to be spatially controlled. It is already known that in vivo an axon from a polarized neuron crosses a complex environment to reach its target. This directionality is critically involved in the degeneration of neurons in the brain^3^. For these reasons, since the seventies many research efforts have been dedicated to design compartmentalized systems useful to study neurite extension of neuronal cells in vitro^4^. In the last couple of decades, microelectronics-derived microfabrication technologies were applied to the study of cell behaviour in vitro, leading to microfluidic cell culture platforms^5^. Nowadays, these represent an established technology for the study of complex cell dynamics in vitro^6^ and the pharmacologic response to specific drugs with unprecedented level of control over a wide number of cell populations and experimental factors^7,8^. Indeed, parameters, such as type and position of cells^9,10^ as well as gradients of specific biomolecules, fluid flow and shear stress^5,11^ can be easily controlled and monitored, leading to significant effects on differentiation, function and long-term survival of several cell types^12,13^. Controlling of multiple cues in the culture microenvironment, which regulate intracellular signalling and cell phenotype, is fundamental for the maintenance and differentiation of stem cells^14,15^. Jackson-Holmes et al. explored key parameters in microfluidic culture of 3D stem cell aggregates trying to understand how these parameters influence stem cell behaviour and differentiation^16^. In particular, they differentiated embryonic stem cells (ESCs) to motor neurons (MNs), assessing how media exchange frequency modulates the biochemical microenvironment, including availability of exogenous factors and cell-secreted molecules, and thereby impacts differentiation. Stem cells (i.e. embryonic stem cells-ESCs-, human-induced pluripotent stem cells-hiPSCs) represent a unique opportunity for biopharmaceuticals, cell-based therapies and personalized medicine since they can be obtained directly from patients and differentiated into multiple cell types^17,18^. In the field of neurobiology research, microfluidic platforms have been developed to study neurite extension of neuronal cells^19,20^ or neurodegenerative diseases^21,22^. Jeon’s group was the first one to fabricate a microfluidic two-compartment device for neuronal cell culture and investigation of axon biology in vitro^23^. Through the years, this design had experienced small variations, such as the introduction of a third cell compartment^19^ or a circular layout^24^. Microfabricated devices have also been developed to differentiate neural stem cells or iPSCs towards motor neurons or glia cells, either as proof-of-concept or drug testing platform^25,26^. In Kim’s^27^ work, NSCs were cultured and differentiated in neurons in response to the concentration gradient of growth factors. In particular, they showed that overexpression of ASCL1 in NSCs increased neuronal differentiation depending on the concentration gradient of growth factors generated in the device, allowing to study concentration-dependent effects of growth factors within a single device. Dittlau et al.^28^ established a versatile and reproducible in vitro model of a human motor unit to study the effect of ALS-causing mutations, generating iPSCs-derived motor neurons and human primary mesoangioblast-derived myotubes in microfluidic devices.

In this study, we developed a three-compartment device that can be used to control cell migration, neurite guidance between different compartments, and cell differentiation on chip. The platform, fabricated by SU-8-based multi-level optical lithography and PDMS replica molding, had three different perfusable compartments with distinct inlets and outlets, interconnected through a series of narrow and parallel microchannels. Using a neuroblastoma cell line (SH-SY5Y) and primary Schwann cells, we tested several geometrical features and different microfluidic setups for controlling cell migration across the microchannels, evaluated the contribution of different adhesion molecules on cell seeding and proliferation, and performed cell differentiation on chip.

## Methods

### Chip design and fabrication

**Figure 1:**
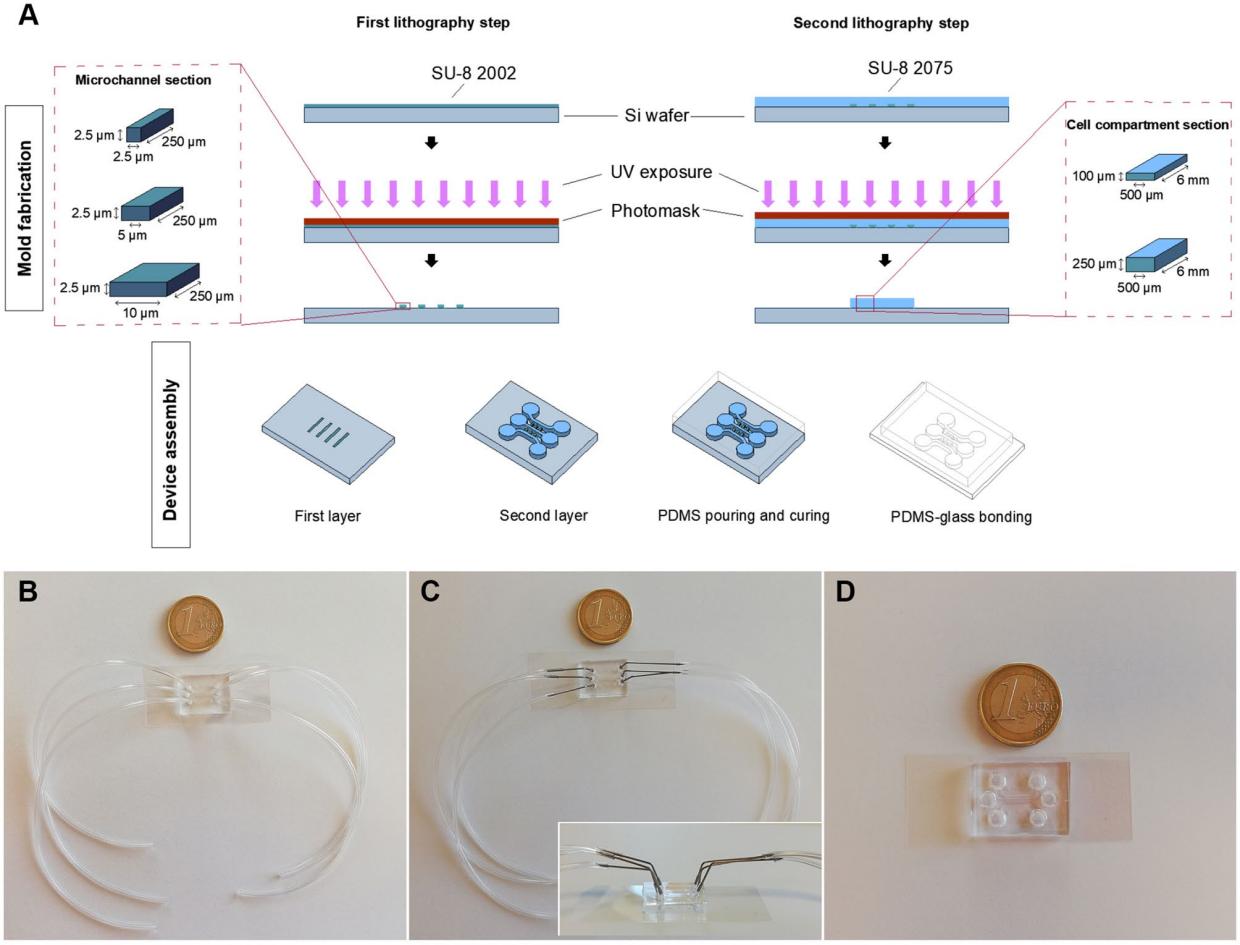
(**A**) Fabrication of the microfluidic device. The mold was realized on a silicon wafer via two-step photolithography process (top side). The final PDMS device was fabricated using soft lithography techniques: PDMS mix was poured on the mold, cured, and peeled off. The replica was sealed on a coverslip by means of oxygen plasma treatment and temperature (bottom side). (**B**–**D**) Chip setups for cell culture. (**B**) Tube system (TS). (**C**) Steel connectors system (SCS). (**D**) Reservoirs system (RS).

The design of the microfluidic chip was inspired by Park’s work^29^. In particular, in Park’s work, the microchannels were 3 µm high and 10 µm wide, while the two main channels were 100 µm high, 1.5 mm wide and 7 mm long. In our work (Fig. 1A, Table S1), the chip consists of three perfusable compartments (500 μm wide, either 100 or 250 μm high and 6 mm long) with distinct inlets and outlets (diameter of 2 mm), interconnected through a series of narrow and parallel microgrooves (either 2.5, 5 or 10 μm wide, 2.5 μm high and 250 μm long) that can allow the separation between soma and neurites and promote unidirectional neurite elongation from one cell compartment to the adjacent one. The microfluidic chip was fabricated via conventional lithography techniques. In particular, the mold was realized via two-step photolithography. First, silicon substrates of 2.5 × 2.5 cm were washed with acetone and isopropanol (Sigma Aldrich, Milan, Italy) and used in a first lithography step to fabricate the microgrooves. A uniform layer of photoresist (SU-8 2002, Kayaku Advanced Materials, Westborough, MA, US) was spin-coated, soft-baked and exposed to UV radiation (λ = 365 nm) using a mask aligner apparatus (MA6, SUSS MicroTec, Garching, Germany), following the manufacturer guidelines. After post-bake, the photoresist was developed in SU-8 developer solution (Kayaku Advanced Materials) and hard-baked. In the second lithography step, SU-8 2075 (Kayaku Advanced Materials) was spin-coated on the substrate to obtain a resist thickness of 250 µm. Following drying and soft-bake, the substrate was exposed to UV radiation, after aligning dedicated markers present on the first level pattern (on the substrate) and the second one (on the photomask) using a mask aligner apparatus. The substrate was post-baked, developed and finally hard-baked. PDMS replicas were later fabricated by soft-lithography using a prepolymer: curing agent (Sylgard 184, Dow, Midland, MI, US) ratio of 10:1. After polymerization, the cured PDMS layer was punched with a 1.7 mm biopsy punch at the inlets and outlets. To create the final devices, microstructured PDMS layers and clean glass coverslips were treated in an oxygen plasma asher (PICO low-pressure plasma system, Diener electronic, Ebhausen, Germany) at 100 W, 200 sccm, for 6 s and heat-treated at 75 °C for 4 h to achieve an irreversible bonding. Some experiments related to the cell adhesion coating assessment were conducted on devices without microgrooves (i.e., no first lithography step was performed) in order to accelerate the fabrication of the devices.

### Cell culture

#### Human neuroblastoma cell line (SH-SY5Y)

Human neuroblastoma cells (SH-SY5Y) were cultured at 37 °C with 5% CO_2_ and expanded in Dulbecco’s modified Eagles medium high glucose (DMEM; Corning, New York, USA), supplemented with 10% (v/v) heat-inactivated fetal bovine serum (FBS; Corning), 1% penicillin–streptomycin (P/S; Corning) and 1% l-glutamine (Corning). Cells were detached from the substrate with Trypsin/EDTA (Corning) and suspended in media at a cellular concentration of 1 × 10^6^ cell/mL before use in further studies on chip. Cells below passage 20 were used in order to avoid cell senescence.

Since SH-SY5Y are neuroblast-like cells, they can be differentiated into a more mature neuronal phenotype using several differentiating agents, including retinoic acid (RA) and neurotrophins (i.e. Brain-derived neurotrophic factor, BDNF)^30,31^. The differentiation method used in this work was developed by Forster et al^32^. The differentiation of SH-SY5Y cells can be divided into two steps using phase 1 and phase 2 media. According to this approach, after 7 days the cells will be differentiated. In particular, the *phase 1 medium* is composed of DMEM (without sodium pyruvate), 5% FBS, 1% P/S, 1% l-glutamine supplemented with 10 µM RA (Santa-Cruz Biotechnology, Dallas, TX, US) included just before the medium is added to the cells. The *phase 2 medium* is composed of Neurobasal-A minus without phenol red (Invitrogen, Carlsbad, San Diego, US), 1% P/S, 1% l-glutamine, 1 × B27 (Gibco), supplemented with BDNF (Biolegend, San Diego, California, US) at a concentration of 50 ng/mL. In our experiment, the cells were pre-differentiated with *phase 1 medium* in Petri dish and then, detached and seeded in the central compartment of the microfluidic device at a cellular concentration of 1 × 10^6^ cells/mL. The cells were maintained in culture on chip for 7 days, under dynamic conditions, using the *phase 2 medium*. The medium was pumped by means of an external programmable syringe pump (NE-4000, New Era Pump Systems, Farmingdale, NY, US) with a flow rate of 0.1 µL/min.

#### Primary rat Schwann cells

All animal procedures were approved by the Internal Ethical Committee of University of Salento, authorized by the Italian Ministry of Health (authorization n° 109/2014-B) and performed according to the current Italian and EU animal welfare legislation and in compliance with ARRIVE guidelines. Pregnant Sprague–Dawley rats were purchased from Harlan Srl and were housed under a 12/12-h light–dark cycle in standard environmental conditions (22 ± 1 °C, 50 ± 5% humidity) with food and water at libitum. The day of birth was counted as postnatal day 0 (P0). Primary Schwann cells were purified from sciatic nerves of male and female newborn rats at P3 (3 days old) as described previously^33^. Briefly, upon isolating from the surrounding muscle and connective tissue, nerves were digested in 0.1% collagenase and 0.25% trypsin at 37 °C for 30 min. Cells were plated onto poly-l-lysine (PLL) coated dishes and, after adhesion, exposed to cytosine arabinoside (Sigma Aldrich) for 48 h to remove mitotic cells, which include most of the fibroblasts. Thereafter, cultures were treated with antiserum to Thy-1 (Bio-Rad AbD Serotec, California, USA) and rabbit complement (Calbiochem, San Diego, USA) to remove remaining fibroblasts. For growth and expansion of primary Schwann cells, cultures were maintained in medium composed of DMEM supplemented with 10% FBS, 5 ng/mL neuregulin (Recombinant Human NRG1-beta 1, R&D Systems, Minnesota, USA) and 2 μM forskolin (Sigma Aldrich). For cell culture on chip, Schwann cells were seeded onto PLL coated devices (0.1 mg/mL in H_2_O) with different microchannels width (2.5 µm, 5 µm and 10 µm) at a cellular concentration of 1 × 10^6^ cells/mL. The cells were monitored using microscopic phase contrast images and, after 48 h, they were fixed and stained with FITC-phalloidin and DAPI to visualize actin cytoskeleton and nuclei, respectively. Schwann cells can be differentiated and induced towards myelinating phenotype using ascorbic acid (AA, Sigma Aldrich)^34^. For this purpose, these cells were seeded onto PLL coated devices at a cellular concentration of 3.5 × 10^5^ cell/mL. After 1 day, the medium, supplemented with 50 µg/mL ascorbic acid, was pumped by means of an external programmable syringe pump with a flow rate of 0.1 µL/min. The cells were maintained in culture on chip for 10 days, under dynamic conditions.

### Setup for cell seeding and culture

In order to optimize the cell seeding step in terms of number of cells present in the device compartments, three chip setups were tested (Fig. 1B–D). In the first setup (Tubing setup, TS, Fig. 1B), Tygon ND 100–80 medical tubing (ID 0.5 mm; OD 1.5 mm) were directly inserted into the inlet/outlet ports, while in the second setup (steel connectors setup, SCS, Fig. 1C) steel connectors were used between the chip and the tubing. Before each experiment, in both cases, all the devices were tested by pumping ultrapure water by means of the syringe pump, in order to verify any leakage out from the chip. In the third setup (reservoirs setup, RS, Fig. 1D), we created circular reservoirs at the level of inlet/outlet ports, punching the PDMS layer with a 4 mm biopsy punch. To test the devices, we filled the inlet reservoirs with 35 µL of ultrapure water each and let the system reach an equilibrium (equal volume in the inlet and outlet reservoirs) by exploiting the concept of communicating vessels. Before plating SH-SY5Y cells, the cell compartments were equilibrated with DMEM and, after the detachment, cells suspended in the same medium and seeded at a density of 1 × 10^6^ cell/mL. In particular, for the SCS and TS approaches, the cellular suspension was injected into the devices through the tubing, while in the RS 50 µL of cellular suspension was placed inside the inlet, refilled with additional DMEM after 3 h. Cell growth at 1 h, 6 h, 24 h and 48 h of SH-SY5Y cells was compared. Values were normalized to cell count measured at 1 h.

### Cell adhesion coating

Different adhesion molecules were tested to coat the cell compartments and select the best growth substrate for culturing SH-SY5Y cells on chip. Before coating, the devices were sterilized in a biosafety cabinet, under UV light, for 1 h. The molecules used in this study were: PLL 0.1 mg/mL in H_2_O, mouse laminin (Santa-Cruz Biotechnology, Dallas, TX, US) 0.01 mg/mL in H_2_O, fibronectin bovine plasma (Sigma Aldrich) 0.01 mg/mL in PBS (Corning), rat collagen (R&D System, Minneapolis, MN, US) 0.25 mg/mL in acetic acid (0.1% v/v in H_2_O). For TS and SCS, the single compartments were filled with 200 µL of coating solution and incubated for 1 h at room temperature. Afterwards, the compartments were rinsed twice with 1 mL DI water and, before cell seeding, filled with 250 µL of cell culture medium. In the RS approach, the inlets were filled with 50 µL of coating solution and, after achieving equilibrium between inlet and outlet of each compartment, all the reservoirs were filled with additional 40 µL. After 1 h incubation, the compartment was rinsed with H_2_O using the same approach.

### Cell staining

Before staining, tubes and steel-connectors were removed from the devices and all the steps were carried out using a P20 pipette connected directly to the inlets. Briefly, cells grown in the compartments were washed with PBS twice to eliminate dead cells and then fixed, at room temperature, with 4% paraformaldehyde in PBS 0.1 M for 15 min. After that, the cells were washed with PBS twice. Cells were permeabilized by exposure to 0.2% Triton X-100 in PBS for 5 min and then two washing steps with PBS were carried out for 3 min each. Later, 1% BSA in PBS was added for 30 min. Primary antibodies anti-β-tubulin III (βtubIII, 1:250, Sigma Aldrich), anti-choline acetyltransferase (ChAT, 1:100, Sigma Aldrich) and anti p75 neurotrophin receptor (1:200, Merck Millipore, Massachusetts, US) were diluted in 1% BSA in PBS and incubated at 4 °C overnight. For the binding of secondary antibodies conjugated with Alexa 488 (1:2000 in 1% BSA in PBS, Thermo Fisher, Milan, Italy) and Alexa 546 (1:1000 in 1% BSA in PBS, Thermo Fisher), samples were washed twice with PBS and then incubated for 1 h at room temperature. Finally, cells were stained with Phalloidin-FITC (1:100, Sigma Aldrich) in 1% BSA in PBS for 20 min and then washed with PBS twice for 3 min and stained with DAPI solution (diluted 1:10,000 in water) for 20 min. Then, the channels were washed twice with PBS for 3 min.

## Results and discussion

### Setup

The design of the microfluidic chip was inspired by Park’s work^29^. Three perfusable compartments (500 μm wide, 100 μm high and 6 mm long) with distinct inlets and outlets (diameter of 2 mm) were interconnected through a series of narrow and parallel microgrooves (either 2.5, 5 or 10 μm wide, 2.5 μm high and 250 μm long). As reported in literature, 450–500 µm microchannel length allows the separation between soma and neurites^35,36^, but even shorter microchannels (200 µm) was sufficient to reach this isolation^37^.

After the device fabrication, we tested different fluidic setups in order to optimize the cell seeding step to ensure the homogeneous distribution of cells along the device compartments and their appropriate growth/survival in the long term. We chose three different approaches as described in "Setup for cell seeding and culture" section. In the TS approach, tubes were directly inserted into the chambers and both static and dynamic cultures conditions could be adopted. The SCS is a modification of the first approach, with the advantage that since the tubes used for cell seeding remain full of cells, the possibility to remove and substitute them represents a benefit to the cells present in the cell compartments, avoiding depletion of nutrients. Moreover, this approach could be useful to easily switch from static to dynamic culture conditions, by disconnecting the tubing from the connector without perturbing the channels (e.g., during medium replacement or administration of a drug). Both TS and SCS represent approaches potentially ready for automation and suitable for long-term experimental designs, although they do not present any control on the flow through the thin microchannel across the cell compartments enabling effective neurite separation. This could be reached by the third approach (RS), that included reservoirs with a diameter of 4 mm. This particular design is very easy to utilize and highly recurrent in literature, also in neuroscience research, as shown by several works^38,39^. It proved indeed to be highly effective in separating somas and neurites when a small volume difference between the reservoirs was introduced, leading to a continuous flow across the microchannels due to a hydrostatic pressure difference^29^. Nevertheless, this approach might be difficult to automate for long-term experiments.

To analyze the effects of the different fluidic setups on cell seeding (Fig. S1, S2), the surface of cell compartments was preliminarily functionalized with PLL, a coating agent commonly used to promote adhesion of various cell types to solid substrates^40^. The use of the TS configuration, without disconnecting the pristine tubing from the chip, allowed the optimal seeding of the cells and an optimal cell attachment. Also, in all the tests conducted using the SCS configuration, it was observed the homogeneous distribution of the cells in the channels and optimal cell attachment. However, this approach had some drawbacks: (1) the replacement of tubes caused the formation/entry of air bubbles into the system, potentially affecting cell survival; (2) if the cells were not completely adhered, all the stresses due to the replacement of the tubes caused their detachment and loss. However, this setup still represents an interesting strategy for more complex dynamic conditions, e.g. when specific on-chip valves are integrated^41^. Finally, also the RS configuration allowed the homogeneous distribution of the cells during the seeding and good cell attachment.

Total cell count was used as a measure of the cell growth in the device compartments subjected to the different fluidic setups (Fig. S3). The number of cells decreased over time in the RS configuration and only few cells remained in the device at 24 and 48 h (compared to the initial number of seeded cells measured 1 h after the seeding procedure). In the other device setups, the cell number/viability remained over 75% up to 24 h. However, at 48 h of culture cell number drastically dropped in all the devices regardless the fluidic setup used. Overall, these observations highlight the need to provide the adequate medium/nutrients replacement to support viability and growth of the cells in the device compartments. Considering the early decrease in the number of cells observed using the RS setup and the drawbacks reported using the SCS configuration, all the following tests were performed adopting the TS setup.

### Coating the device with cell adhesion molecules

**Figure 2:**
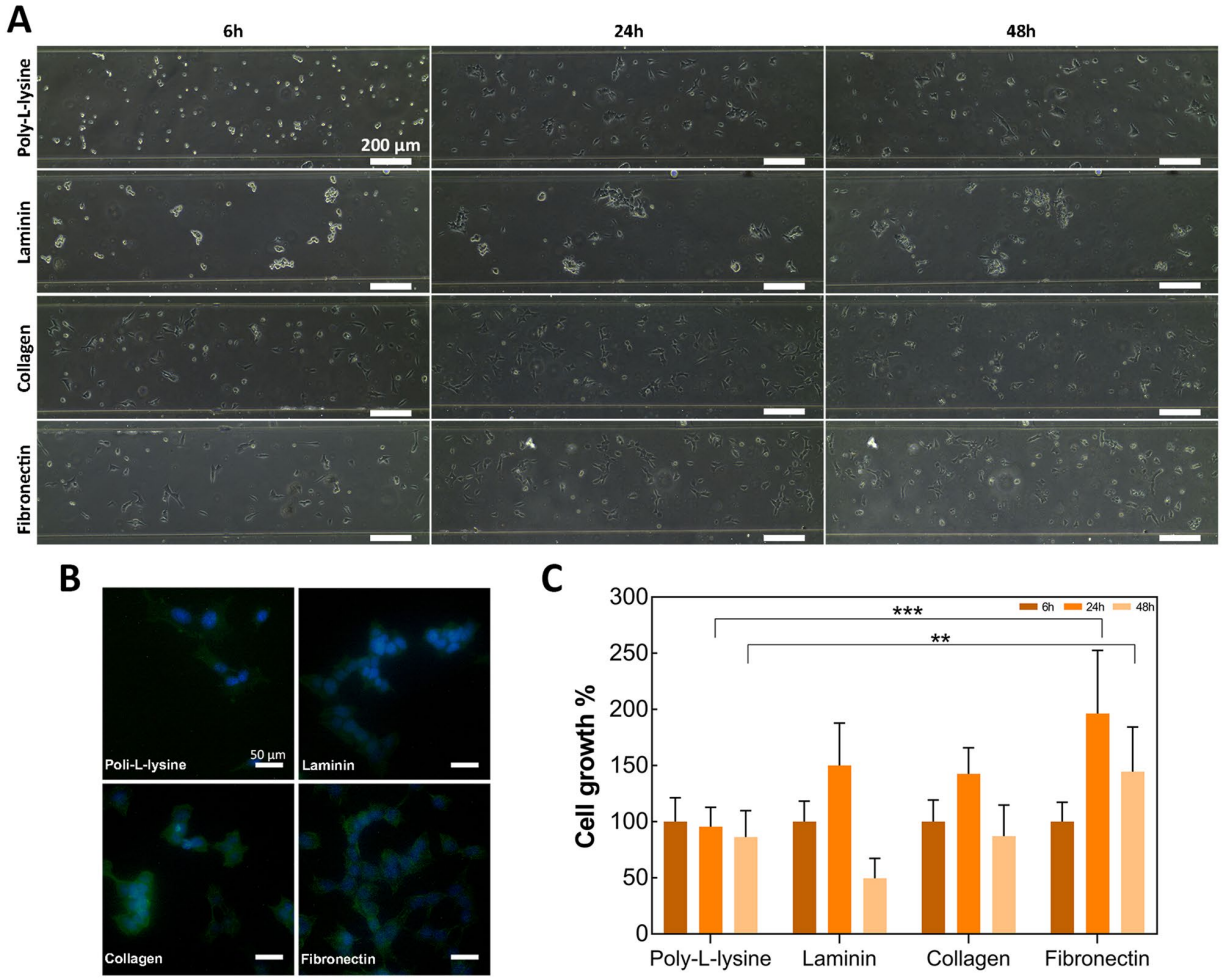
Cell behavior using different adhesion molecules. (**A**) Phase contrast microscope images of SH-SY5Y cells, grown on different substrates in microfluidic devices at 6, 24, and 48 h. Scale bar: 200 μm. (**B**) Fluorescent images of SH-SY5Y cells. DAPI and Phalloidin-FITC stained SH-SY5Y cells grown on different substrates in microfluidic devices at 48 h. Scale bar: 50 μm. (**C**) Bar graph representation of SH-SY5Y cell growth cultured on different substrates at 6, 24 and 48 h, following normalization to cell count measured at 6 h. Histogram bars represent the average of 3 experiments and error bars indicate the standard deviation (Two-way ANOVA test: **p < 0.005; ***p < 0.001).

Collagen, fibronectin and laminin are natural components of the extracellular matrix (ECM) of the nervous system^42,43^ that are commonly used in cell culture to promote cell adhesion. These proteins were investigated as adhesion substrates for the microfluidic device, and compared to PLL as coating agents. Cell attachment and growth were monitored over a period of 48 h through optical microscopy (Fig. 2A,B, S4). SH-SY5Y cells were able to attach and grow on all four surfaces. Cells cultured on PLL and laminin exhibited a more spherically-shaped morphology and formed cell clusters. On the other hand, SH-SY5Y cells grown on collagen and fibronectin showed rapid cell attachment (i.e. cells attached within 6 h) and exhibited a flat, epithelial-like morphology with few short processes^30^. Notably, cell number increased culturing SH-SY5Y on chips coated with each of the different ECM proteins compared to PLL-functionalized chips (Fig. 2C), with fibronectin leading to the greatest cell number. According to literature, fibronectin supports adhesion of various cell types, as well as also neurite outgrowth from developing both central and peripheral nervous system neurons^44–46^ and it is used for culturing SH-SY5Y on microfluidic device^13^. However, after 48 h of cell culture the number of cells decreased, regardless the coating molecule under evaluation, likely due to nutrient depletion in the culture medium as it was not replaced throughout the experiments. Overall, these data define fibronectin, among all the substrates evaluated, as the best substrate to functionalize the devices for obtaining high cell proliferation. For this reason, all the following tests were performed using this molecule as coating molecule.

### Effect of the cell compartment geometry (height) on cell viability

**Figure 3:**
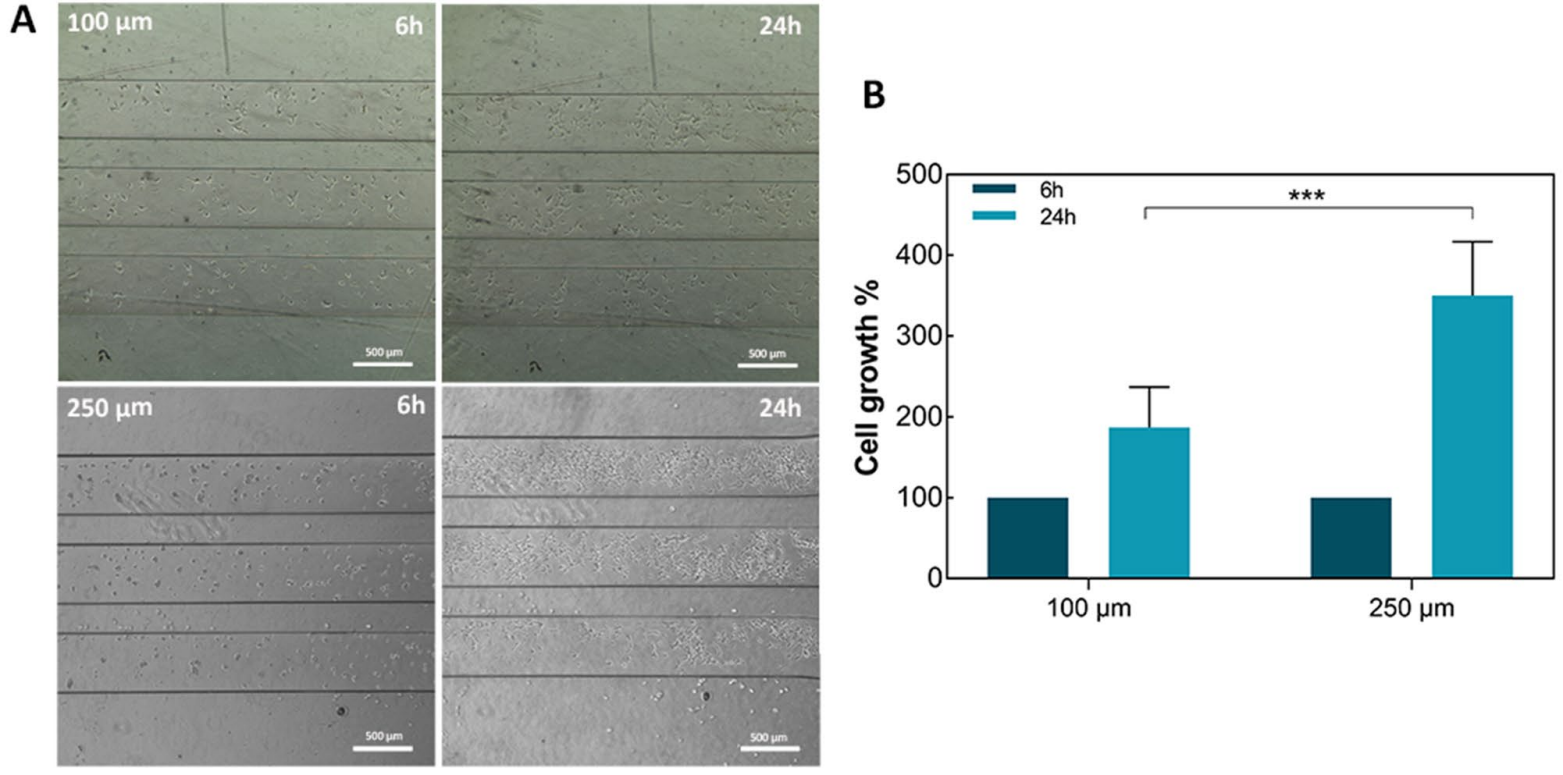
Effects of different channel heights on cell growth. (**A**) Phase contrast microscope images of SH-SY5Y grown on microfluidic device having 100 μm and 250 μm channels at 6 h and 24 h. Scale bar: 500 μm. **(B**) Bar graph representation of SH-SY5Y cell at 6 and 24 h, following normalization to cell count measured at 6 h. Histogram bars represent the average of 4 experiments and error bars indicate the standard deviation (Two-way ANOVA test: ***p < 0.001).

To mitigate the negative effects on cell survival/growth due to the depletion of nutrients from the cell medium occurring in the long-term cell culture, we increased the height of the cell compartments to 250 µm with the aim to supply the cells with a larger amount of medium/nutrients and simultaneously decrease the shear stress inside the channels. Devices with 100 µm- and 250 µm-high channels and without microgrooves were seeded with 1 × 10^6^ cells/mL and the seeding density/cell number (measured at 6 h) in the 250 µm-high channels was 2.1 times higher than in 100 µm-high channels. The SH-SY5Y cells seeded in 250 µm-high reached cell confluence in 24 h and exhibited a statistically significant increase in cell number/growth compared to cells seeded in 100 µm-high channels (Fig. 3).

### Geometry-driven control of cell migration

**Figure 4:**
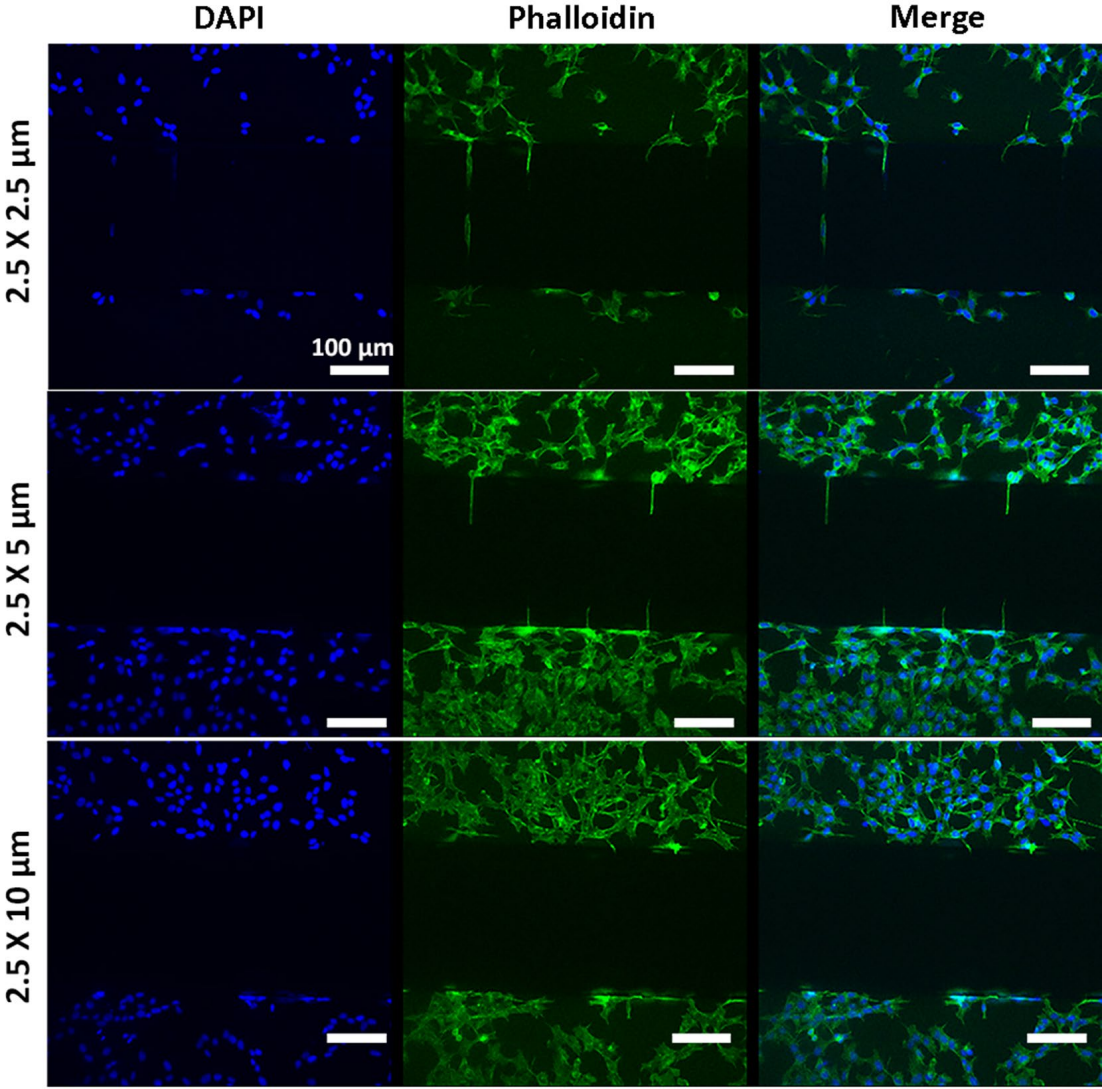
Fluorescent images of SH-SHY5Y cells cultured on devices having microchannels with different height × width section. DAPI and Phalloidin-FITC stained SH-SY5Y showed how only 2.5 × 5 µm geometry permitted neurite elongation, preventing soma migration. Scale bar: 100 µm.

**Figure 5:**
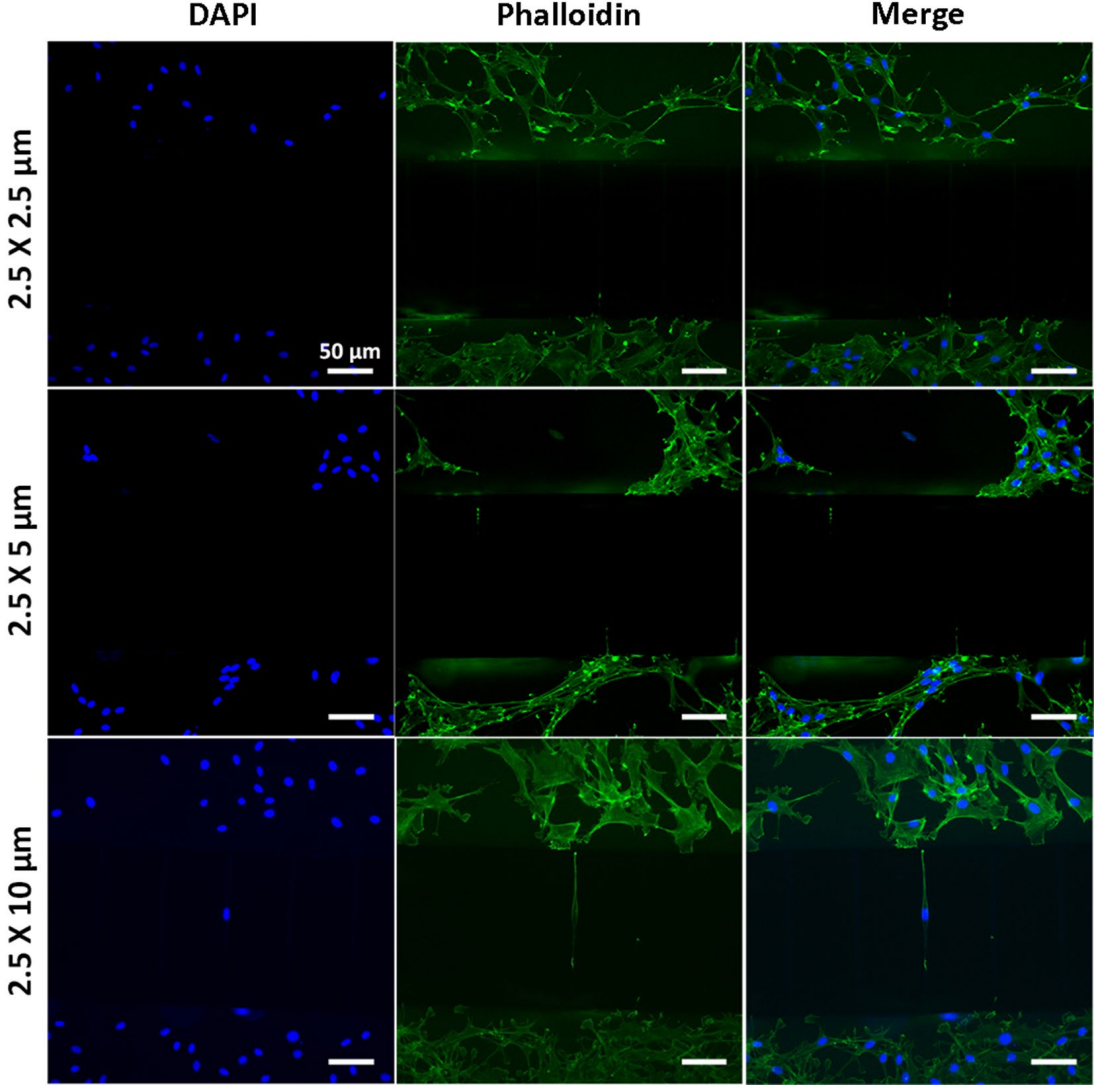
Fluorescent images of primary rat Schwann cells cultured on devices having microchannels with different height × width section. DAPI and phalloidin-FITC stained primary rat Schwann cells showed how only 2.5 × 5 µm geometry permitted cell protrusions, preventing nuclei migration. Scale bar: 50 µm.

The separation of neurites from their somas is an essential requisition to study axon guidance phenomena or neuronal degeneration. For this purpose, various groups investigated different microchannel height (2–4 µm)^24,47^, width (2–10 µm)^19^ and length (35–900 µm)^19,48,49^. Based on these studies, we designed devices with 250 µm-high perfusable compartments connected through 61 microchannels, having different width (2.5, 5 and 10 µm) while keeping constant height (2.5 µm) and length (250 µm) (Fig. 1A). SH-SY5Y cells were seeded on devices with different microchannel geometry, cultured for 48 h, fixed and stained with FITC-phalloidin and DAPI to visualize actin cytoskeleton and nuclei, respectively (Fig. 4). Microchannels of 2.5 × 10 µm allowed the migration/passage of both the cell nucleus and cytoplasm. Contrarily, 2.5 × 5 µm geometry only allowed the elongation of cell processes/neurites, preventing cells/nuclei migration and providing an optimal platform to study axons-mediated cell–cell communication. Finally, microchannels of 2.5 × 2.5 µm did not allow either nuclear migration or cytoskeletal elongation, likely preserving soluble factors-mediated communication only. Similar results were obtained when primary rat Schwann cells were cultured onto PLL coated devices (Fig. 5). Thus, the latter geometry could be a powerful tool for focusing primarily on paracrine effects.

### On chip differentiation of SH-SY5Y and primary rat Schwann cells in flow condition

**Figure 6:**
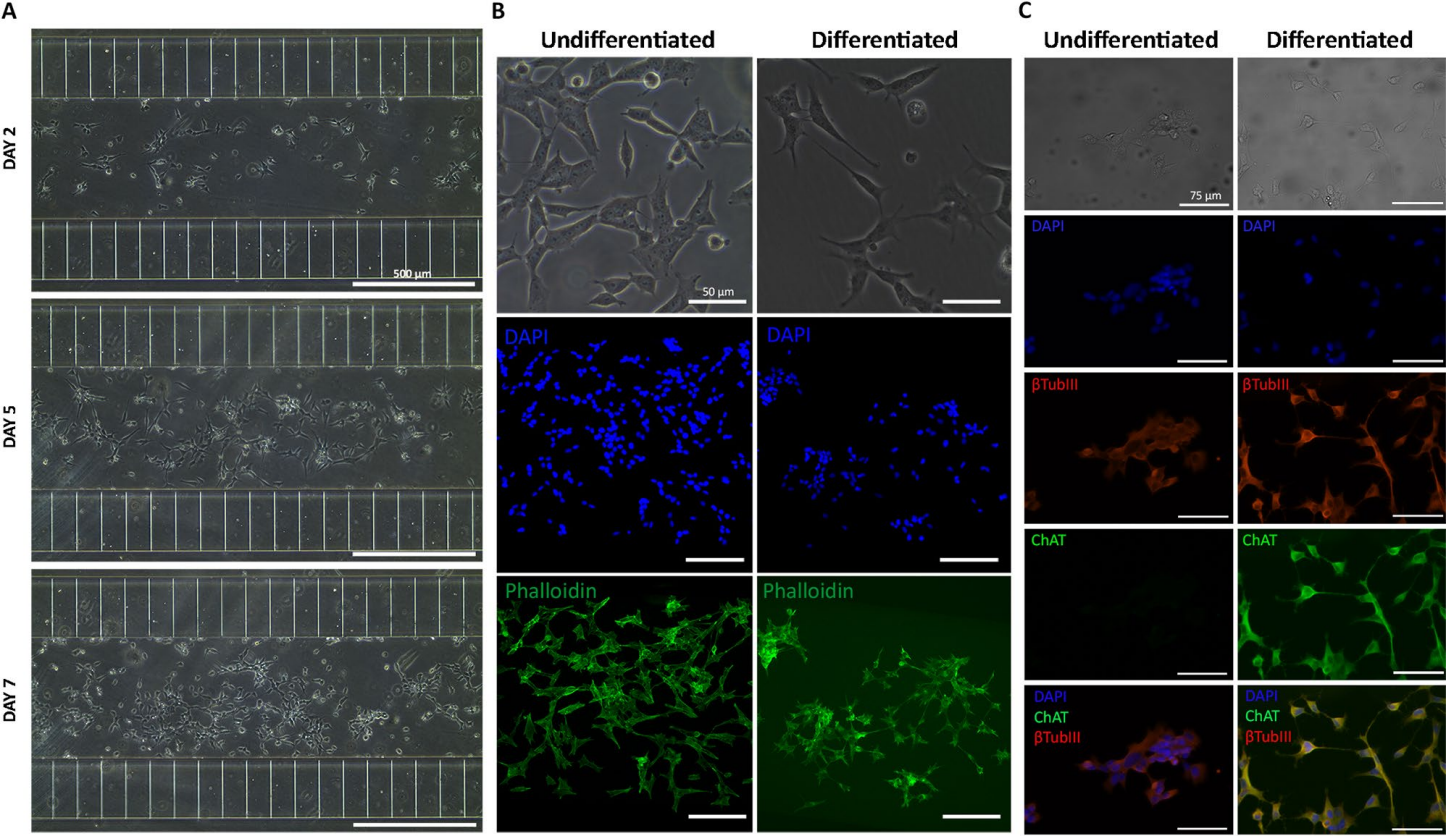
Differentiation of SH-SY5Y cells in microfluidic device in flow condition. (**A**) Phase contrast images of SH-SY5Y under differentiation at day2, day5 and day7 of incubation with *phase 2 medium*. Scale bar 500 µm. (**B**) Phase contrast and fluorescent (DAPI/phalloidin) images of undifferentiated SH-SY5Y cells and differentiated cells after 7 days of incubation. Differentiated SH-SY5Y became morphologically more similar to primary neurons with a more pyramidal shaped cell body and long and numerous but randomly distributed processes. Scale bar: 50 µm. (**C**) Fluorescent images of βtubIII/ChAT stained undifferentiated and differentiated SH-SY5Y cells. Scale bar: 75 µm.

We explored the possibility to use the devices in a long-term/dynamic experiment, adapting a protocol to differentiate SH-SY5Y and primary rat Schwann cells directly on chip. SH-SY5Y cells can be differentiated toward cholinergic, adrenergic, or dopaminergic phenotype employing various differentiation agents and media conditions^30,50^. In Forster’s protocol, SH-SY5Y cells undergo firstly to FBS starvation and RA treatment, followed by exposure to BDNF. As shown in literature, RA is the most commonly agent/treatment used to induce the differentiation of these cells towards a cholinergic neuronal phenotype^51–53^ and BDNF treatment promotes the conversion of undifferentiated SH-SY5Y cells that acquire distinct neuron-like features^54^. Pre-differentiated SH-SY5Y cells were seeded on devices and maintained for 7 days in differentiation medium under flow condition (0.1 µL/min). The differentiation was monitored using microscopic phase contrast images and at the end of the differentiation procedure cells were fixed and stained with FITC-phalloidin, DAPI and neuron markers, βtubIII and ChAT (Fig. 6). Contrarily to wild-type SH-SY5Y cells, SH-SY5Y cells differentiated on chip acquired the primary neurons-like morphology with a pyramidal shaped cell body and long, numerous and randomly distributed cell processes/neurites^51^. Moreover, in undifferentiated SH-SY5Y cells, ChAT expression was negligible as expected, since this enzyme is a specific marker of cholinergic (differentiated) neurons^55^. Overall, these data highlight the possibility to use this microfluidic setup and these devices as a tool for on chip differentiation studies extended over several days, without the need of media replacement and/or any substantial decrease in nutrients concentration.

**Figure 7:**
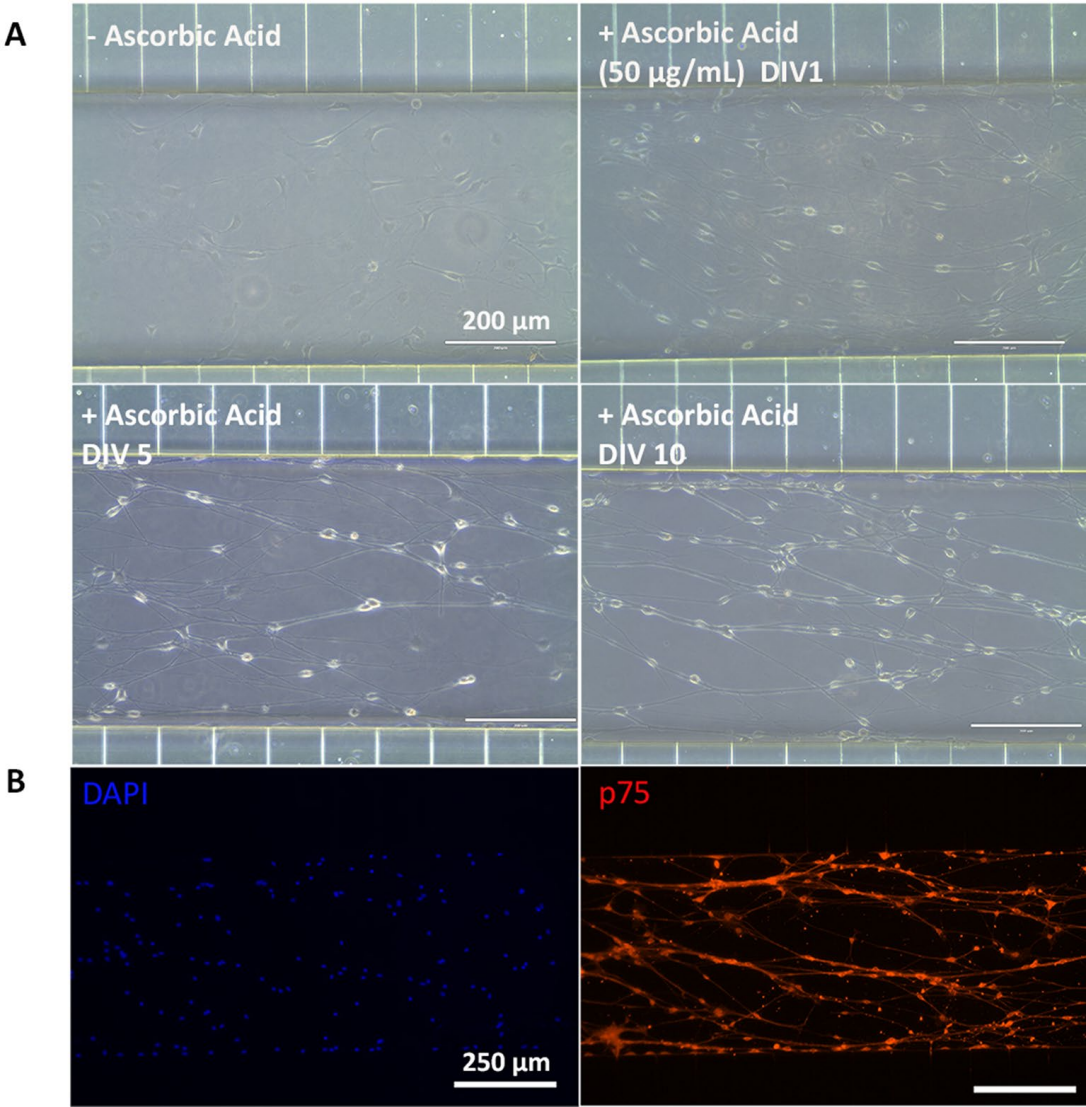
Induction of primary rat Schwann cells towards a myelinating phenotype in microfluidic device in flow condition. (**A**) Phase contrast images of primary rat Schwann cells before treatment with ascorbic acid and under differentiation at DIV1, DIV5 and DIV10 of incubation with ascorbic acid (50 µg/mL). Scale bar 200 µm. (**B**) Fluorescent images of p75 neurotrophin receptor p75 stained induced primary rat Schwann cells at DIV10. Scale bar: 250 µm.

Schwann cells (SCs) play key roles in development, differentiation, physiological homeostasis, and axonal regeneration in the peripheral nervous system. Moreover, they are responsible for providing trophic support for the growth and maintenance of neurons producing myelin sheath^56^. As reported in literature, ascorbic acid is widely used as a myelination-inducible factor^34,57^. Therefore, as described previously, Schwann cells were maintained for 10 days with myelinating medium containing 50 µg/mL ascorbic acid and the induction was monitored using microscopic phase contrast images (Fig. 7A). After 10 days, cells were fixed and stained for p75 neurotrophin receptor (Fig. 7B), that is a neurotrophin receptor involved in a diverse array of cellular responses, including apoptosis, neurite outgrowth and myelination^58^. Phase contrast and fluorescent images show how SCs had fine cell body, assuming the slender, elongated morphology typical of Schwann cells and expressing p75 neurotrophin receptor^59,60^.

## Conclusions

In summary, we developed a PDMS-based multi-compartment microfluidic device useful for the study of neuron-neuron interactions in vitro as a powerful tool in neuroscience research. In particular, a protocol was established in order to microfabricate a microfluidic chip by multi-level optical lithography. Moreover, different microfluidic setups (tubes system, steel connectors system and reservoirs systems) were evaluated in order to analyze their effect on cell seeding. The results showed that, among the different setup used, TS configuration leads to optimal seeding and cell attachment, with a homogeneous cell distribution and morphology in the channels and it was chosen to perform all the following test. Secondly, collagen, fibronectin and laminin were tested as cell adhesion molecules. Fibronectin allowed a rapid cell attachment and proliferation, displaying SH-SY5Y cells with a flat, epithelial-like morphology and few short processes. Next, different macrochannels height (100–250 µm) and different microchannel section (2.5, 5 and 10 µm) were tested to evaluate the influence of the geometry on the cell viability and neurite elongation. Data showed that 250 µm-high macrochannels and 2.5-high and 5 µm-wide microchannels allowed an increase in cell seeding and the elongation of cell neurites, preventing nuclei migration for both cell types used in this work. Moreover, the device was used in long-term experiments in order to differentiated SH-SY5Y and primary rat Schwann cells, towards a more mature neuron-like phenotype and myelinating phenotype, respectively. Further studies will be focusing towards the use of such devices with more physiologically relevant cells, such as neural stem cells or induced-pluripotent stem cells. Moreover, we envision that our platform will be useful for further studies in neurobiology, where co-culturing different cell types (i.e. neuron, Schwann cells, muscles) in distinct compartments is needed in order to investigate physiopathological mechanisms behind neurodegenerative diseases.

## Supplementary Information

Supplementary Information.
