# Supplementary material for: A microfabricated multi-compartment device for neuron and Schwann cell differentiation
Source: Sci Rep. 2021 Mar 29;11:7019. doi: 10.1038/s41598-021-86300-4 (PMC8007719; doi:10.1038/s41598-021-86300-4)
Supplement: Supplementary file 1 — Supplementary Information. [file 41598_2021_86300_MOESM1_ESM.pdf]

Supplementary information for

A microfabricated multi-compartment device for neuron and Schwann cell differentiation

Eleonora De Vitis[1,2], Velia La Pesa[3], Francesca Gervaso[1*], Alessandro Romano[3], Angelo Quattrini[3], Giuseppe Gigli[1,2], Lorenzo Moroni[1,4], Alessandro Polini[1*]

[1] CNR NANOTEC – Institute of Nanotechnology, Campus Ecotekne, via Monteroni, Lecce, 73100, Italy.
[2] Università Del Salento, Dipartimento di Matematica e Fisica E. de Giorgi, Campus Ecotekne, via Monteroni, Lecce, 73100, Italy
[3] IRCCS San Raffaele Scientific Institute, Division of Neuroscience, Institute of Experimental Neurology, Milan, 20132, Italy
[4] Maastricht University, Complex Tissue Regeneration, Universiteitssingel 40, Maastricht, 6229 ER, Netherlands

Email: francesca.gervaso@nanotec.cnr.it; alessandro.polini@nanotec.cnr.it

Results and discussion

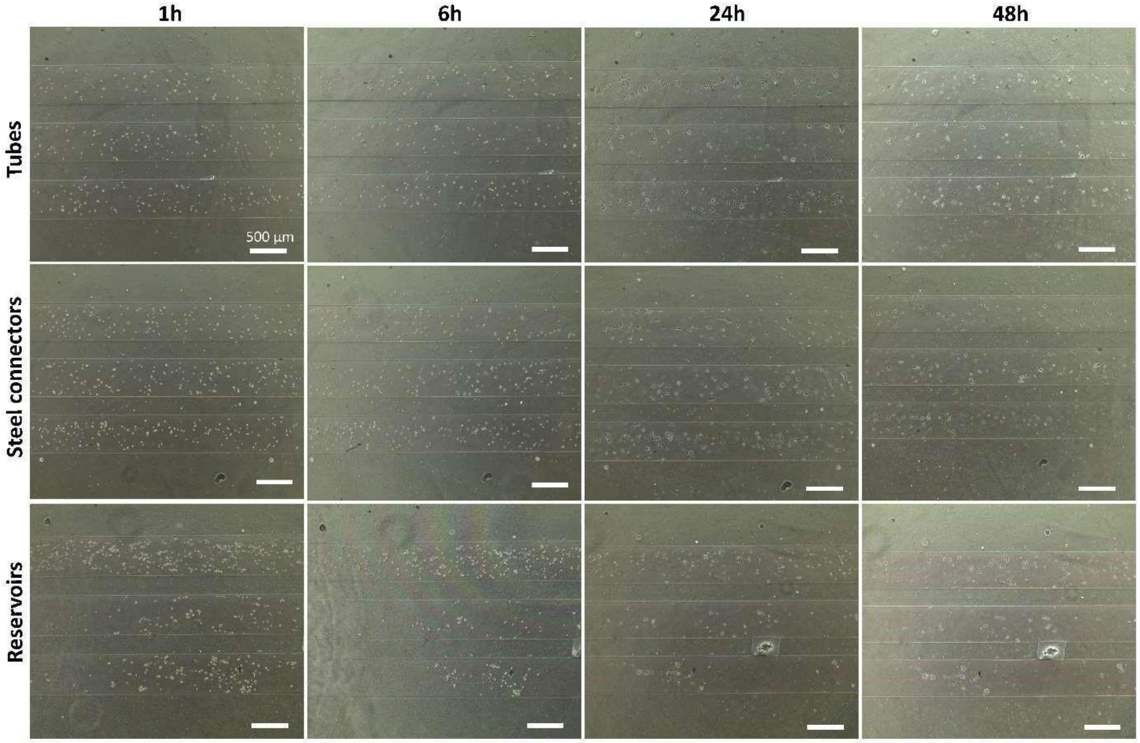

Figure S1: Optical investigation of cell growth. Phase contrast images of SH-SY5Y cells seeded in microfluidic devices using different fluidic setups. Scale bar = 500µm.

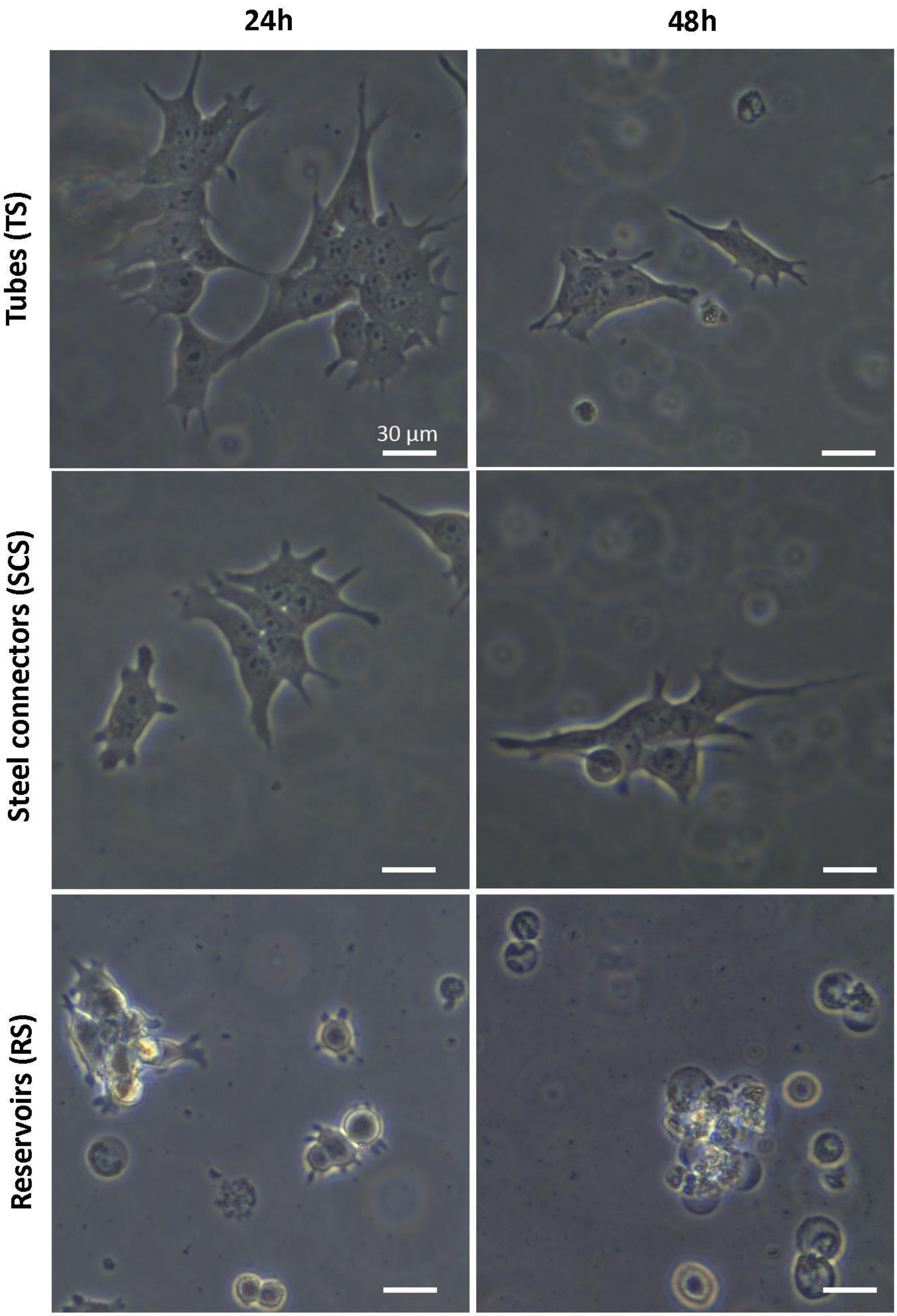

Figure S2: SH-SY5Y cells grown in microfluidic devices. Phase contrast microscope images of SH-SY5Y cells, at 24h and 48h, seeded in microfluidic devices using different fluidic setups. Scale bar = 30µm.

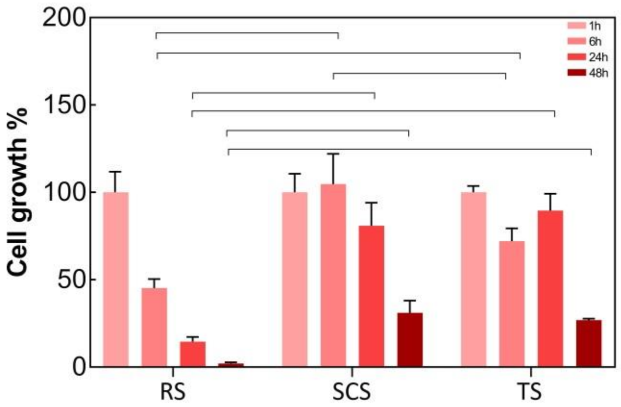

Figure S3: Cell growth analysis. Bar graph representation of SH-SY5Y cell growth at 1, 6, 24 and 48 hours, following normalization to cell count measured at 6h. Histogram bars represent the average of 3 experiments and error bars indicate the standard deviation (Two-way ANOVA test: $p<0.001$).

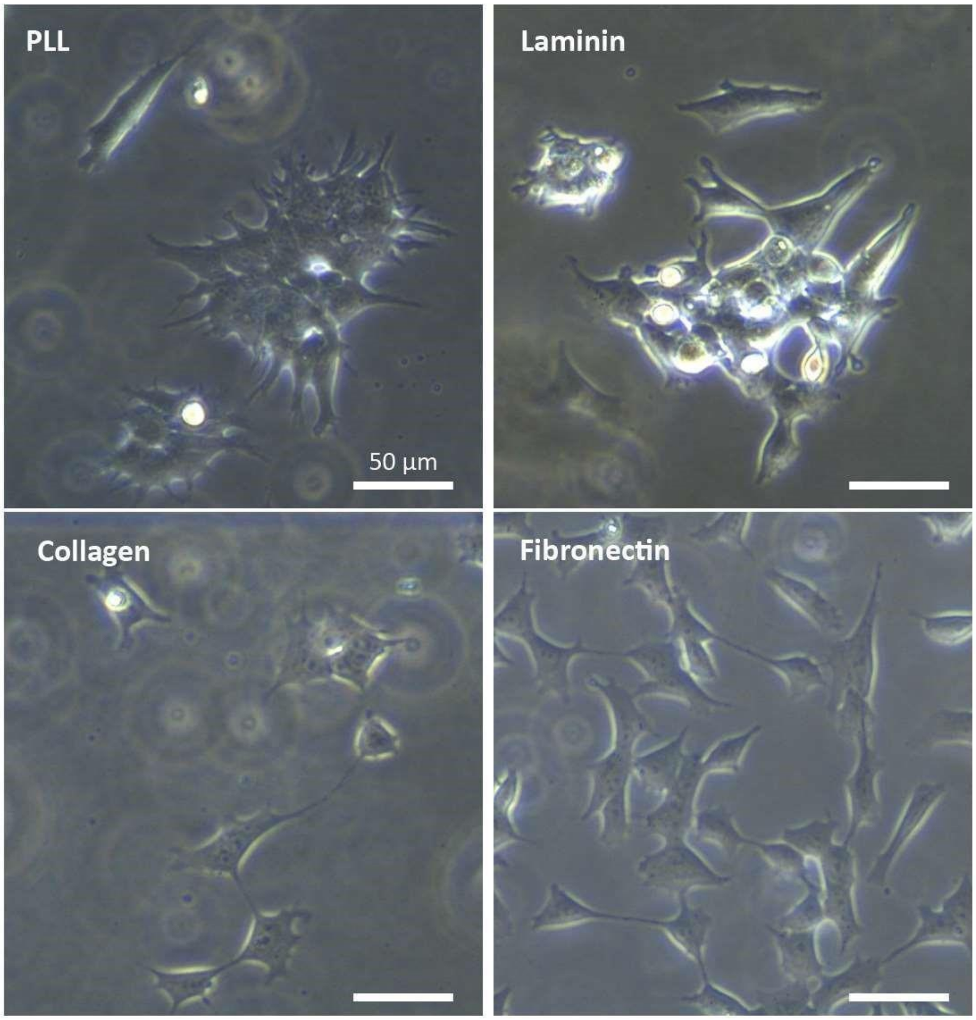

Figure S4: On chip cell growth comparison. Phase contrast microscope images of SH-SY5Y cells, grown on different substrates in microfluidic devices at 24 hours. Scale bar: 50µm.

	Cell compartments				Microchannels			
	n	*Width (µm)*	*Length (µm)*	*Height (µm)*	*n*	*Width (µm)*	*Length (µm)*	*Height (µm)*
**Park's device**	2	1500	7000	100	120	10	150	3
**Our device**	3	500	6000	100-250	61	2.5-5-10	250	2.5

Table S1: Features comparison between Park's[1] and our device.

[1] Park, J.W., et al., *Microfluidic culture platform for neuroscience research.* Nat Protoc, 2006. **1**(4): p. 2128-36.
